# Stability Matters: Revealing Causal Roles of G-Quadruplexes (G4s) in Regulation of Chromatin and Transcription

**DOI:** 10.3390/genes16101231

**Published:** 2025-10-17

**Authors:** Ke Xiao, Rongxin Zhang, Tiantong Tao, Huiling Shu, Hao Huang, Xiao Sun, Jing Tu

**Affiliations:** 1 State Key Laboratory of Digital Medical Engineering, School of Biological Science and Medical Engineering, Southeast University, Nanjing 211189, China; kexiao@seu.edu.cn (K.X.); xsun@seu.edu.cn (X.S.); 2 Robert Lurie Comprehensive Cancer Center, Department of Obstetrics and Gynecology, Feinberg School of Medicine, Northwestern University, Chicago, IL 60611, USA

**Keywords:** G-quadruplex, thermostability, causal Bayesian network, regulation of chromatin and transcription

## Abstract

Background: G-quadruplexes (G4s) are non-canonical higher-order nucleic acid structures that form at guanine-rich motifs, with features spanning both secondary and tertiary structural levels. These dynamic structures play pivotal roles in diverse cellular processes. Endogenous G4s (eG4s) function through their dynamically formed structures, prompting the hypothesis that their thermostability, as a key structural property, may critically influence their functionality. This study investigates the relationship between G4 stability and other functional genomic signals within eG4 regions and examines its broader impact on chromatin organization and transcriptional regulation. Methods: We developed a mapping strategy to associate in vitro-derived thermostability metrics and multi-omics functional signals with eG4 regions. A stability-centric analytical framework combining correlation analysis and causal inference using the Bayesian networks was applied to decipher causal relationships between G4 stability and the other related signals. We further analyzed the association between the stability of transcription start site (TSS)-proximal eG4s and the biological functions of their downstream genes. Results: Our analyses demonstrate that G4 thermostability exerts causal effects on epigenetic states and transcription factor binding, thereby influencing chromatin and transcription regulation. We further show distinct network architectures for G4-binding versus non-binding transcription factors. Additionally, we find that TSS-proximal eG4s are enriched in genes involved in core proliferation and stress-response pathways, suggesting that eG4s may serve as regulatory elements facilitating rapid stress responses through genome-wide coordination. Conclusions: These findings establish thermostability—though measured in vitro—as an intrinsic property that shapes eG4 functionality. Our study not only provides novel insights into the functional relevance of G4 thermostability but also introduces a generalizable framework for high-throughput G4 data interpretation, significantly advancing the functional decoding of eG4s across biological contexts.

## 1. Introduction

G-quadruplexes (G4s) are non-canonical four-stranded structures formed by guanine-rich sequences typically characterized by a classic consensus motif G_3+_N_1−7_G_3+_N_1−7_G_3+_N_1−7_G_3+_ [1], though it is now recognized that G4 formation can occur through more diverse sequence arrangements [2,3,4]. Within a G4 structure, guanine bases form planar G-tetrads via Hoogsteen hydrogen bonding, and the G-tetrads stack on each other [5]. Genomic DNA G4s participate in diverse biological processes and are implicated in human diseases [6,7,8], positioning them as potential therapeutic targets, particularly in cancer research.

Identifying genomic G4 loci is essential for functional studies. Putative G4s (pG4s) are typically predicted using motif-based algorithms like G4Hunter [3] and pqsfinder [4]. However, only a subset of pG4s can form genuine structures [9]. To address this, methods such as G4-seq [9] and G4-miner [10] detect G4 formation in vitro by quantifying thermostability (hereafter “stability”) via abnormal quality of high-throughput sequencing data. Regions identified in vitro are termed observed G4s (oG4s). Endogenously formed G4s (eG4s) are further identified in vivo using antibody-based techniques (e.g., ChIP-Seq, CUT&Tag) with high-affinity antibodies (e.g., BG4, G4P) [11,12,13,14], revealing up to tens of thousands of eG4 structures across cell lines.

eG4s are implicated in gene regulation and chromatin remodeling [15,16]. They localize to nucleosome-depleted regions [11,13], colocalize with REV1 polymerase [17,18], and are associated with epigenetic marks (e.g., histone acetylation, methylation) [19,20], suggesting roles in shaping the epigenetic landscape. Genome-wide studies also suggested eG4s as binding hubs for transcription factors (TFs) [21]. Nevertheless, unresolved mechanistic questions persist: since eG4s function as structures, how do structural properties of eG4s influence these associated processes? Critically, what quantifiable features can characterize the structural determinants governing eG4 activity?

We hypothesize that eG4 stability, which reflects the capacity to maintain structural integrity, intrinsically influences eG4 interactions with chromatin environment, thus regulating chromatin dynamics and transcription. This influence remains underexplored: while Chambers et al. [9] proposed in vitro mismatch percentage (MM%) from sequencing mistakes caused by G4-induced polymerase stalling, as a stability metric for oG4 screening, regions exceeding the threshold were indiscriminately treated as oG4s, and differences in stability were not further analyzed. In the first comprehensive study of G4 stability distribution, Guiblet et al. [22] delineated stability distributions across genic regions, correlating them with G4 function and selective pressure. However, the causal impact of eG4 stability on biological processes, especially regulation of chromatin and transcription, has yet to be established.

**Figure 1:**
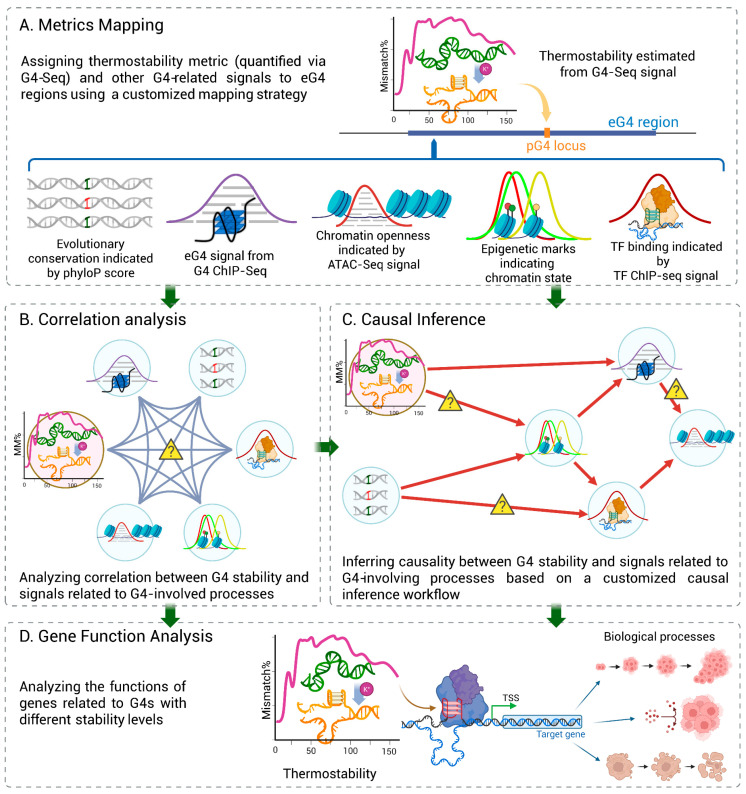
The framework for stability-centric functional analysis. (**A**) Structural stability metrics mapped to eG4 regions, alongside eG4 signal from G4 ChIP-Seq data, evolutionary conservation indicated by phyloP score, and other metrics about chromatin/transcriptional regulation, including chromatin openness indicated by ATAC-seq signal, chromatin states indicated by epigenetic marks, and TF-binding events inferred from TF ChIP-Seq data. (**B**) Correlation analysis between these G4-related metrics. (**C**) Causal inference among these signals. (**D**) Functional analysis of genes related to G4s with different stability levels, TSS-proximal G4s, and the functions of the downstream genes was analyzed. Created with BioRender.com.

Herein, we employ MM% as an experimentally determined metric of structural stability and mapped it to eG4 regions. Alongside eG4 stability, we also incorporate metrics reflecting (1) in vivo G4 formation, (2) evolutionary conservation, and (3) chromatin/transcriptional regulation—including chromatin accessibility, epigenetic states, and TF occupancy. Through correlation analysis and causal inference, we decipher the relationship between these metrics and demonstrate causal effects of stability on these functional correlates. Furthermore, based on gene function analysis, we suggest that the stability of eG4s proximal to transcription start sites (TSS) relates with the downstream gene functions, and highly stable eG4s may orchestrate functional programs involving the downstream genes. We thus posit eG4 stability as a fundamental factor of G4-involving processes and provide a framework for stability-centric functional analysis (Figure 1).

## 2. Materials and Methods

### 2.1. Data Availability

All used datasets are summarized in Table 1.

### 2.2. Identification eG4 Regions

G4 ChIP-Seq data of K562 and HepG were obtained from Spiegel et al. [21] and processed by a modified workflow based on the one of Hansel-Hertsch et al. [11], with hg19 as the reference. The runs from the same biological replicate were combined, so we have three replicates for each cell line. After peak calling by MACS2 (version 2.2.7.1) [23], with a *p*-value cutoff of $10^{-4}$ in SE mode, the consensus regions containing over two overlapping peaks were ranked and filtered by MSPC (version 6.0.0) [24], with the weak and stringency thresholds set as $10^{-4}$ and $10^{-8}$, respectively. The G4 ChIP-Seq data of HEK293T were obtained from Li et al. [13] and processed using their workflow. Peaks were called by MACS2 in PE mode with the same *p*-value cutoff, and the consensus regions were also processed by MSPC, which were defined as those containing either one peak or two overlapping peaks from the two replicates. Consensus regions with *p*-values above the cutoff were defined as eG4 regions, and only eG4 regions containing at least one pG4 motif were kept for further analysis. For each region, the score generated by MSPC based on combined *p*-value was employed as the intensity of eG4 sequencing signal.

### 2.3. Generating and Mapping Stability Levels

The G4-Seq data for the Na^+^ vs. K^+^ conditions were obtained from Chambers et al. [9] and processed by the workflow they proposed. G4 thermostability was quantified using the mismatch percentage (MM%) metric, which reflects differential polymerase stalling induced by G4 structures under K^+^ vs. Na^+^ conditions. Since the enhanced stability observed specifically in the presence of K^+^, this K^+^-dependent signal provides a close in vitro approximation of G4 stability under near-physiological ionic conditions.

The pG4 loci were generated by pqsfinder (v2.0.1) [4]. For each pG4 locus, the maximum MM% values from the same strand within a 150 bp window centered on the locus were assigned to the pG4. The MM% value of the pG4 that is closest to the midpoint of the region was assigned to the region as the stability level.

### 2.4. Mapping Chromatin States to eG4 Regions

The 18-state model were downloaded from the Roadmap Epigenomics Project [25], which was trained by using signals of six histone modifications, H3K4me1, H3K4me3, H3K9me3, H3K27ac, H3K27me3, and H3K36me3, from 111 reference epigenomes. The predicted chromatin states for K562 and HepG2 were also obtained from the project [25]. For HEK293T, the H3K4me1, H3K4me3, and H3K27ac data were from Li et al. [13], H3K9me3 from Zhang et al. [26], H3K27me3 from Gene Expression Omnibus (GEO) with accession ID GSE235014, and H3K36me3 from Bhattacharya et al. [27]. The chromatin states were then predicted by the 18-state model based on the epigenetic marks.

For all the three cell lines, the state of the midpoint from each eG4 region was assigned as the chromatin state of the latter.

### 2.5. Mapping ATAC-Seq Intensity to eG4 Regions

ATAC-Seq data for K562 and HepG2 were obtained from ENCODE portal with accession ID ENCSR483RKN and ENCLB750JRI, respectively, and ATAC-Seq data for HEK293T were from GEO with accession ID GSE235014. The reads were aligned to hg19 by using bowtie2 (version 2.3.5.1) [28], with both alignments for the individual mates and discordant alignments disabled, in the end-to-end and very-sensitive mode. The aligned reads were then piled up by MACS2 in PE mode. The average coverage of these fragments in each eG4 region was normalized with the average sequencing depth and then regarded as the ATAC-Seq signal intensity of the region.

### 2.6. Mapping phyloP Score to eG4 Regions

The human referenced phyloP scores were obtained from the Zoonomia Project, which was generated from 241 mammalian genomes [29]. For each pG4 locus, the average phyloP score within a 50 bp window centered on the locus was regarded as the phyloP score for that pG4. Following the same strategy for mapping stability to eG4s, phyloP scores were then assigned to each of the eG4 regions.

### 2.7. Count the Occupancy of TFs in eG4 Regions

The TF-binding peak regions for all three cell lines were extracted from the ReMap2022 non-redundant dataset [30]. For an eG4 region, the occupancy metric, i.e., the hits of a specific TF, was defined as the number of overlapping peaks, and the TF recruitment metric, i.e., the number of colocalized TFs, was defined as the amount of the hits within this region.

### 2.8. Workflow of Bayesian Network Construction

**Figure 2:**
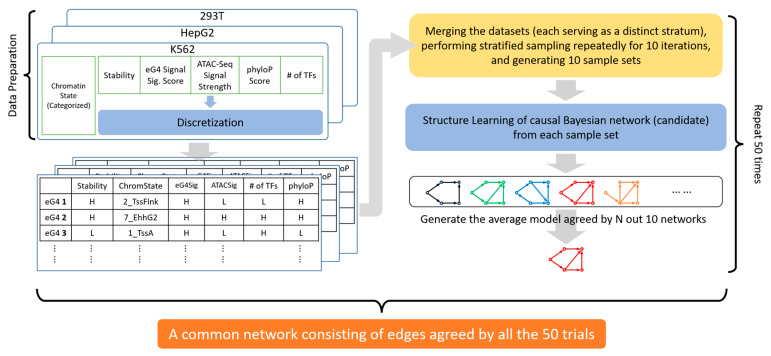
Workflow of structure learning of a causal Bayesian network. The stratified sampling strategy (yellow rectangle) and the threshold N for generating the common network are adjustable.

The causal Bayesian networks containing stability level, eG4 signal significance score, phyloP score, ATAC-seq signal intensity, chromatin state, and the number of colocalized TFs within eG4 regions were constructed by a customized workflow (Figure 2). All numeric metrics, except the stability level, were discretized (“High”/”Low”) based on cell-line–specific median values, while stability metric was discretized by using an empirical cutoff of 25. To mitigate potential bias arising from the substantial variation in the number of detectable eG4 regions across cell lines (K562: 7639; HepG2: 21,996; HEK293T: 8986; Tables S1–S3)—even when processed through identical analytical pipelines—which may stem from inherent biological differences or technical variability in sample preparation, we merged all genomic metrics from K562, HepG2, and HEK293T. We then applied stratified sampling, with cell lines as distinct strata, to capture the cell-type–independent relationships among these signals. Two strategies were tested: proportional-allocation, extracting fixed percentage per stratum, and equal-allocation, with fixed sample count per stratum, as well as different sampling sizes.

For each combination of strategy and sampling size, we performed 10 sampling iterations. Causal structures were learned from each sample set as candidates, and an average model was then defined as agreed by N of the 10 sample sets. As there is no golden standard for the causal relationship among these G4-related signals, we followed the method proposed by Yu et al. [31] and employed the overlap of the candidates to the common network as a surrogate for robustness measurement of the structure. Robustness was quantified via accuracy and coverage: the former is defined as the proportion of consensus edges in candidate networks, and the latter is the averaged ratio of the consensus edges that were identified by a candidate network.

We performed the sampling procedure 50 times and repeated the structure learning on the 10 sample sets of each trial to derive average and standard deviation of the accuracy and coverage. Overall, when the models’ coverage is plotted against their accuracy, the robustness was indicated by the area under the curves (AUC).

### 2.9. Explanation of Metrics and Scores Used in the Workflow

The following metrics and scores were used throughout our analytical workflow:

• Mismatch percentage (MM%) was used to quantify G4 stability by comparing base-calling discrepancies between reads generated under K^+^ and Na^+^ conditions, calculated as the fraction of different calls across the reads [9].
• eG4 signal intensity was represented by the reproducibility significance score generated by MSPC [24], derived from combined *p*-values across biological replicates.
• Chromatin state annotations were generated using the ChromHMM model [25], based on six histone modifications, H3K4me1, H3K4me3, H3K9me3, H3K27ac, H3K27me3, and H3K36me3.
• Chromatin accessibility (ATAC-Seq signal intensity) was measured as the normalized average coverage of ATAC-Seq fragments within each eG4 region.
• Evolutionary conservation (phyloP score) was derived from multiple alignments of 241 mammalian genomes, provided by the Zoonomia Project [29].
• The recruitment of TFs within an eG4 region was evaluated by the total number of TFs colocalized within the eG4 region, and the TFs were from the ReMap2022 non-redundant dataset [30]. The occupancy of a specific TF in an eG4 region was defined as the hits of the TF in the region.

### 2.10. Bayesian Network Inference

We used the bnlearn package (version 5.1) [32] for structure learning of the causal Bayesian networks. A modified PC-algorithm [33] was employed for learning the network (represented as a Directed Acyclic Graph, DAG) from observational data by systematically testing conditional independencies between variables. In a DAG, three canonical structures, i.e., chain (X→Z→Y), fork (X←Z→Y), and collider (X→Z←Y), determine independence [34], where the arrows represent putative causal influences based on conditional independence tests. Here, two sets of nodes, A and B, are defined as d-separated by a set S if and only if every path between any node in A and any node in B is blocked by S, where a path is blocked by S if it contains a chain ( →Z→) or fork ( ←Z→) where Z is in S, or contains a collider ( →Z←) where Z is not in S and no descendant of Z is in S; then A and B are conditionally independent given S ( A⫫B∣S) in any probability distribution faithful to the DAG. Based on the d-separation criterion, the PC-algorithm starts with all possible connections in a graph, removes edges if variables are conditionally independent, finds collider patterns, and propagates directions while avoiding cycles [35]. And the modified algorithm [33] is based on the PC algorithm and resolves the order-dependence in the estimation of the skeleton of a DAG.

In our workflow (Figure 2), we employed the Monte Carlo permutation test of Pearson’s $\chi^{2}$ for conditional independence [36] in the modified PC-algorithm. The algorithm was applied to the 10 sample sets generated by the stratified sampling procedure and then wrapped in the 50-repeat loop.

### 2.11. The Stratified Sampling Strategies

We merged data from all three cell lines and employed stratified sampling, with each cell line serving as a distinct stratum. The proportional-allocation procedures sampling k% (k = 10, 20, 30, 40, 50) samples (i.e., eG4 regions) from each stratum, and the equal-allocation procedures sampling k (k = 1000–7000) samples from each stratum were tested. Because there are fewer than 8000 eG4 regions in K562, the upper limits of sampling number were set as 50% and 7000, respectively, to keep the final sample set sizes comparable.

The performance of the models was compared based on AUC of the accuracy and coverage, and the strategies and sample sizes corresponding to the robust average model, as well as the optimal N value, were used for the generation of the common network, of which the edges were shared by all the 50 trials (average models).

Since the common networks were derived from the outputs of bootstrapping, they might contain bidirectional edges with uncertainty. To resolve these, we retained only the direction with higher empirical frequency. In the cases where both directions occurred with equal frequency, the edge was considered undirected to reflect this uncertainty. Furthermore, in networks modeling selective transcription factor binding, any edge pointing to the G4 stability node was also conservatively treated as undirected to avoid logically inconsistent causal interpretations.

### 2.12. Gene Ontology Enrichment Analysis

We performed Gene Ontology (GO) enrichment analysis of biological processes using the clusterProfiler R package (version 3.0.4) [37] with the org.Hs.eg.db annotation database (v3.21.0). Significantly enriched terms were identified based on the default threshold (0.05) of adjusted *p*-value. For visualization and interpretation, the top 20 most significantly enriched terms per group were displayed.

## 3. Results

### 3.1. G4 Stability Correlates with eG4 Signals as Well as Chromatin and Transcriptional Regulatory Signals

To investigate associations between eG4 structural stability and G4-involving biological processes, we developed a mapping strategy linking eG4 regions—defined as consensus ChIP-Seq peaks from multiple replicates—to stability as well as other related signals (Figure 1A). We analyzed eG4 regions in K562, HepG2, and HEK293T cell lines, focusing exclusively on signals within them. Each eG4 region was attributed to the nearest pG4 to its center, and eG4 stability was then quantified as the maximum G4-Seq MM% value within a 150 bp window centered on the pG4 and restricted to the same DNA strand. Since the G4-Seq data were obtained under the Na^+^ vs. K^+^ conditions [9], this K^+^-dependent metric provides a close in vitro approximation of G4 stability under near-physiological ionic conditions. In this way, we mapped the stability metric to the central pG4 sites and then to the eG4 regions. We also mapped the significance score of antibody-based eG4 sequencing signals, which reflects in vivo G4 structure capture (henceforth termed “eG4 signals”), and the phyloP score, which measures evolutionary conservation at individual alignment sites, to the eG4 regions. Furthermore, metrics about chromatin/transcriptional regulation, including chromatin openness indicated by ATAC-seq signal intensity, chromatin states inferred by ChromHMM as composite epigenetic profiles [25], and the number of colocalized TFs within eG4 regions were taken into consideration.

**Figure 3:**
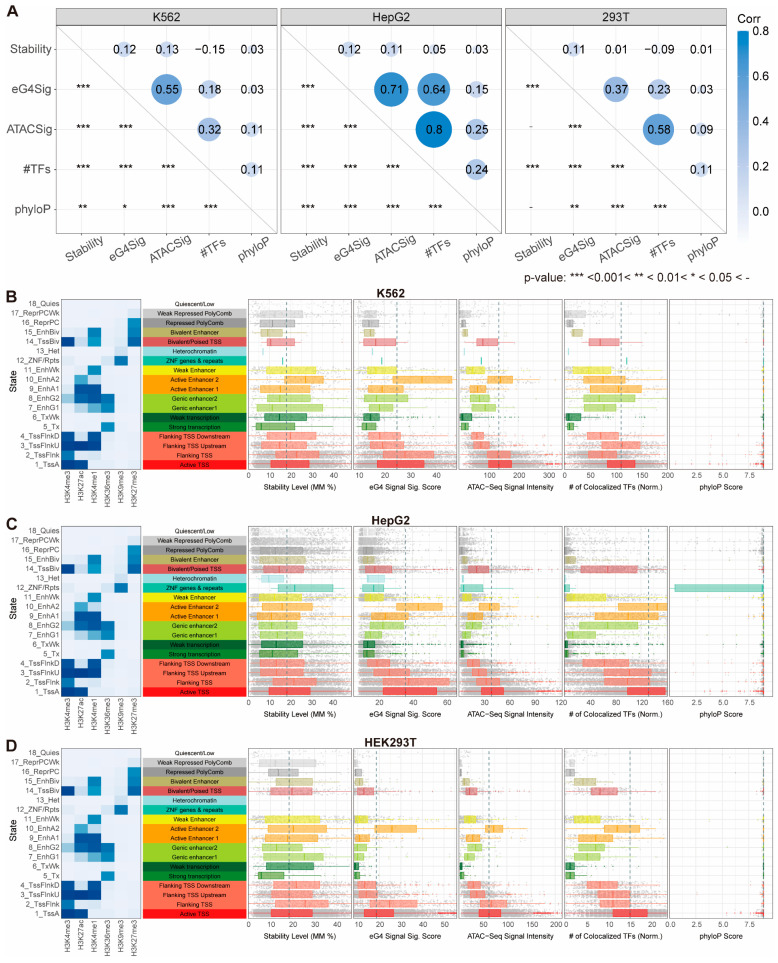
The associations between stability and other G4-related metrics within eG4 regions. (**A**) Spearman correlation coefficients between the stability level, eG4 signal significance score, ATAC-Seq signal intensity, number of colocalized TFs, and phyloP score. The estimated ρ and *p*-values are shown. (**B**–**D**) The distributions of the stability level, eG4 signal significance score, ATAC-Seq signal intensity, number of colocalized TFs (normalized by the maximum), and phyloP score across the chromatin states in K562 (**B**), HepG2 (**C**), and HEK293T (D), respectively. The states were generated by the ‘expanded’ 18-state model published previously [25]. The vertical dashed lines indicate the medians of these metrics from the first state (Active TSS).

We examined associations between these metrics. Spearman correlation analysis (Figure 3A) revealed consistently strong relationships between eG4 and ATAC-seq signals across the three cell lines (ρ=0.55, 0.71, 0.37 for K562, HepG2, and HEK293T, respectively; p<0.001), confirming the established link between eG4 formation and chromatin accessibility [11] while demonstrating quantitative signal interdependence. Similarly, robust correlations emerged between ATAC-seq signal and the number of colocalized TFs (ρ=0.32, 0.8, 0.58;p<0.001), indicating this significant connection between chromatin openness and transcription factor (TF) binding persists within eG4 regions. Unexpectedly, stability exhibited no correlation with eG4 signal (ρ=0.12, 0.12, 0.11), ATAC-seq signal ( ρ=0.13, 0.11, 0.01, respectively), and TF number (ρ=−0.15, 0.05, −0.09). This may reflect either limitations of our stability hypothesis or uncharacterized nonlinear relationships between G4 stability and functional metrics that are difficult to capture by linear correlation.

In contrast, stability distributions varied significantly across chromatin states in all cell lines, revealing strong associations between G4 stability and epigenetic marks within eG4 regions (Columns 1–3 in Figure 3B–D and Figure S1). While demonstrating no direct relationship with individual histone modifications, G4 stability exhibited complex relationships with specific mark combinations. Medium-to-high H3K4me3 levels (Flanking TSS) corresponded to highest overall levels of G4 stability, and intermediate G4 stability persisted in regions marked by concurrent H3K4me3/H3K27ac enrichment (Active TSS). However, triple-mark states of H3K4me3/H3K27ac/H3K4me1 (Flanking TSS Upstream) showed reduced stability, generally lower than Active TSS, while moderate H3K27ac with weak H3K4me1 (Active Enhancer 2) paradoxically corresponded to elevated stability again. These patterns demonstrate that G4 stability relates to histone modifications through combinatorial regulatory logic rather than linear associations.

Intriguingly, parallel—though not perfectly synchronized—trends emerged for G4 stability and eG4 signals, ATAC-Seq signals, and the number of colocalized TFs, with high overall values at Active TSS, Flanking TSS, and Active Enhancer 2 states, and low values at Weak/Strong transcription and (Weak) Repressed PolyComb states (Columns 3–6 in Figure 3B–D). Given the moderate to strong correlations observed among eG4 signals, ATAC-seq signals, and TF-binding number (Figure 3A), these co-variations may arise from multiple causal structures: chromatin state may act as a common cause influencing all three signals, or alternatively, as a mediator within the causal pathways between them. Furthermore, since G4 stability is a sequence-intrinsic property measured in vitro, its partial concordance with the other signals might be interpreted as the influence of G4 stability on the other G4-involving processes mediated by the chromatin state, i.e., the combinatorial pattern of the epigenetic marks. Other unmeasured confounders or more complex causal relationships may also play a role. These hypotheses require further investigation.

Guiblet et al. [22] proposed that evolutionary selection shapes G4 stability at pG4 loci. To evaluate whether selection pressure could confound the observed distributional patterns, we analyzed phyloP scores of G4-forming sequences as indicators of evolutionary conservation. phyloP scores showed negligible correlation with stability (ρ≈0) and minimal correlation to eG4 signals, while demonstrating weak associations with ATAC-Seq signal (ρ=0.09−0.25) and TF number (ρ=0.11−0.24) (Figure 3A). Crucially, phyloP scores remained high across all chromatin states (Columns 7 in Figure 3B–D), arguing against selection pressure as a common driver of the observed relationship between stability and the other G4-related signals. Furthermore, eG4 regions exhibited higher conservation than pG4 motifs (Figure S2), with phyloP scores from pG4s showing greater variance and chromatin-state/genomic-region bias, which underscores the functional importance of endogenously formed G4 structures over motif presence alone. This high conservation of eG4 regions also echoes the conservation of pG4s in regulatory regions reported by Mohanty et al. [38].

### 3.2. Causal Bayesian Network Demonstrates That G4 Stability Affects Other G4-Related Signals

**Figure 4:**
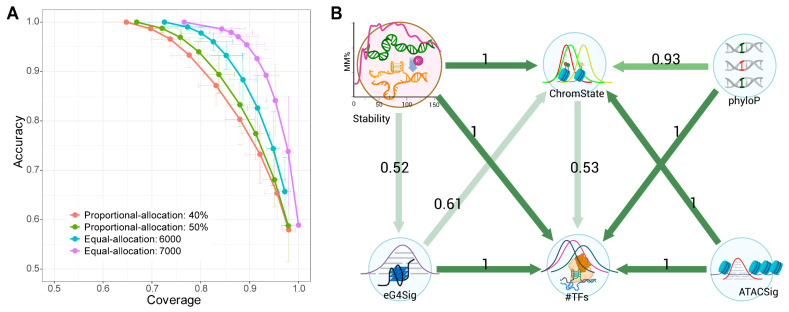
Causal relationship between G4 stability and the other metrics. (**A**) Robustness measurement of models derived from proportional-allocation (extracting 40% or 50% samples per stratum) and equal-allocation (extracting 6000 or 7000 samples per stratum) strategies. The coverage is plotted against accuracy, and the vertical and horizontal bars on the curves indicate the standard deviations of accuracy and coverage. (**B**) The causal Bayesian network exhibiting causal relationship between G4 stability, chromatin state, eG4 signal significance score, ATAC-Seq signal intensity, number of colocalized TFs, and phyloP score, within eG4 regions. The numbers and colors indicate the direction probabilities of the edges. The graph was manually created with BioRender.com, based on the adjacency matrix in Table S4.

To delineate causal relationships beyond correlation, we constructed Bayesian the networks integrating G4 stability, eG4 signal, phyloP score, chromatin state, ATAC-seq signal, and number of colocalized TFs via a customized causal inference workflow (detailed in Methods). To eliminate cell-specific bias and capture the cell-type-independent relationships among these signals, data from K562, HepG2, and HEK293T were discretized (“High”/”Low” relative to cell-type-specific medians) and then merged. We employed stratified sampling, with cell lines as strata, to generate sample sets for the construction of the causal Bayesian networks. Multiple sampling strategies and sizes were tested. Given the absence of golden standard about the causal relationship among these G4-related signals, we adapted Yu et al.’s approach [31], utilizing accuracy and coverage to quantify robustness of the network structures. The “Equal-allocation” strategy, extracting 7000 samples per stratum, demonstrated optimal performance (Figure 4A and Figure S3), and the models closest to the upper right corner of the plot, namely, the networks with edges agreed by five of the 10 models (N=5), were adopted. A common network with edges shared by all the 50 trials/models was then generated (Figure 4B).

The patterns, Stability→ChromState←phyloP and Stability→#TFs←phyloP, demonstrated that both G4 stability and evolutionary conservation independently influence chromatin states and TF-binding events.

Critically, the path from G4 stability to chromatin states—which integrate six histone modification signals—is supported by experimental evidence demonstrating that G4 structures can recruit histone modifiers [39,40,41] and show genomic colocalization with marks such as H3K4me3 and H3K27ac [11,42]. Similarly, the path from G4 stability to TF recruitment is corroborated by both direct affinity pull-down assays and genome-wide enrichment analyses [21,41]. Together, these findings not only confirm the dependency of histone modifiers and TF recruitment on G4 structures but also further reveal that G4 thermostability directly modulates the efficiency of both histone modification machinery and transcription factor recruitment.

In addition to G4 stability and evolutionary conservation, ChromState was also influenced by ATACSig, which aligns with an established causal sequence: ATP-dependent chromatin remodeling complexes are recruited, leading to nucleosome ejection and the formation of broader accessible DNA regions, which subsequently facilitate the deposition of active histone marks [43]. Although the influence of histone modifications on chromatin accessibility—such as acetylation neutralizing lysine charges to promote an open chromatin state [44], or broad H3K4me3 domains serving as platforms for transcriptional machinery and thus enhancing chromatin accessibility [45]—is well documented, the path ATACSig→ChromState here supports the role of chromatin accessibility as a driver of epigenetic state modification. It is important to note, however, that due to the acyclic constraint of the Bayesian networks, such bidirectional regulatory relationships may remain undetected in one direction.

The recruitment of TFs within eG4 regions was also affected by both chromatin openness (via ATACSig→#TFs) and histone modifications (via ChromState→#TFs). This is consistent with the established model, wherein chromatin remodeling and histone modifications create a stable, permissive environment where the opened and marked chromatin landscape then allows conventional, non-pioneer transcription factors to bind to their previously inaccessible DNA regions [46,47].

Furthermore, the direct causal path Stability→eG4s indicates that G4 stability enhances eG4 signal detectability through immediate structural effects. This is supported by the positive correlation between stability and eG4 signal (Figure 3A), reinforcing the conclusion that high stability improves antibody-based detection of G4 structures in vivo.

The proposed causality Stability/phyloP/ATACSig →{ChromState, #TFs}, as well as ChromState →#TFs, demonstrates remarkable consistency across sample sizes and sampling strategies (Figure 4 and Figure S4), suggesting the robustness of the causal paths. Meanwhile, the persistent absence of a Stability-phyloP edge reinforces their mutual independence within eG4 regions. Notably, stability invariably emerged as a cause (never effect) of other signals related to G4-invovling processes, affirming its role as an intrinsic sequence-encoded property. Thus, correlations involving stability likely reflect genuine causal relationships. Given its derivation from in vitro G4-seq data, stability measurement represents a practical starting point for probing G4-related functional impact.

### 3.3. G4 Stability Drives Selective TF Binding

Building upon evidence that eG4s can serve as transcriptional hubs [21], we postulated that their structural stability modulates interactions with TFs, which selectively bind G4s. We therefore extended our Bayesian network model to infer causal relationships, within eG4 regions, among G4 stability, phyloP score, chromatin state, ATAC-seq signal, and occupancy of six distinct TFs. These TFs were selected based on experimentally validated G4-binding properties: SP1, SP2, and YY1 represent established G4-binding factors with demonstrated structural selectivity; TARDBP exhibits ambiguous binding behavior toward both folded G4s and unfolded oligomers, while CTCF and FOXA1, which lack G4-binding capacity, serve as negative controls [21].

**Figure 5:**
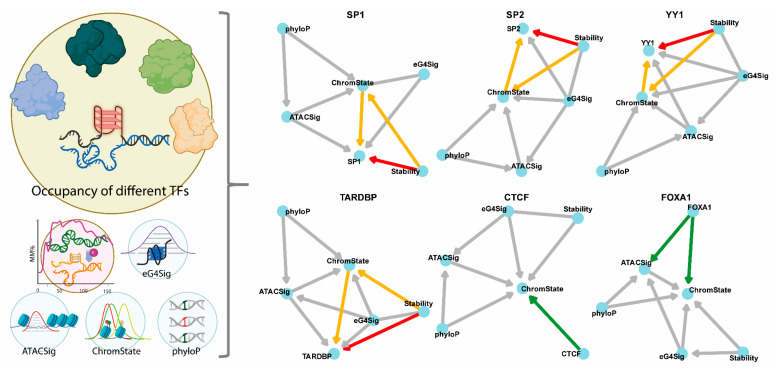
Causal relationships between G4 stability and occupancy of distinct TFs. The Bayesian networks modeling causal interactions among G4 stability, phyloP score, chromatin state, chromatin accessibility, and occupancy of the six TFs: established G4-binders (SP1, SP2, YY1), ambiguous binders (TARDBP), and non-binders (CTCF, FOXA1). The red edges indicate direct effects from G4 stability to occupancy of the TFs, the orange ones indicate indirect effects mediated by ChromState, and the green ones indicate effects from TFs to chromatin openness or histone modifications. Created with BioRender.com.

The resultant network topology (Figure 5) of the canonical G4-binders, SP1, SP2, and YY1, and the ambiguous binder TARDBP, consistently demonstrated causal paths from stability to TF occupancy within eG4 regions, both directly and indirectly mediated via ChromState. This pattern aligns with our intuition and the mechanistic hypothesis that G4 stability modulates affinity between eG4s and these G4-binding TFs.

Conversely, networks for negative controls CTCF and FOXA1 showed no path from G4 stability to TF occupancy. This stark contrast validates the specificity of our approach: causal relationships between G4 stability and TF binding emerge exclusively for genuine G4-interacting factors, while networks for non-binders yield the null hypothesis. Moreover, the causal network correctly captured the effect of CTCF on histone modifications (ChromState), supported by the evidence that CTCF might help to recruit the Polycomb repressive complexes and thus influence the deposition of H3K27me3 [48]. The effect of FoxA1 on chromatin accessibility (ATACSig) and histone modifications (ChromState) was also captured correctly, as FoxA1, a pioneer TF, can bind closed chromatin, initiate chromatin opening, and directly recruit histone-modifying enzymes [46,49].

### 3.4. Stability of the TSS-Proximal eG4s Relates to the Functions of the Downstream Genes

Given the established effects of G4 stability on chromatin environment, including epigenetic states, transcription factor binding, and accessibility within eG4 regions, we also investigated the functional relationships between TSS-proximal eG4s and their downstream genes, focusing on eG4s within ±200 bp of TSSs. Here, stability levels and eG4 signal intensities were binarized (“Low”/”High”).

**Figure 6:**
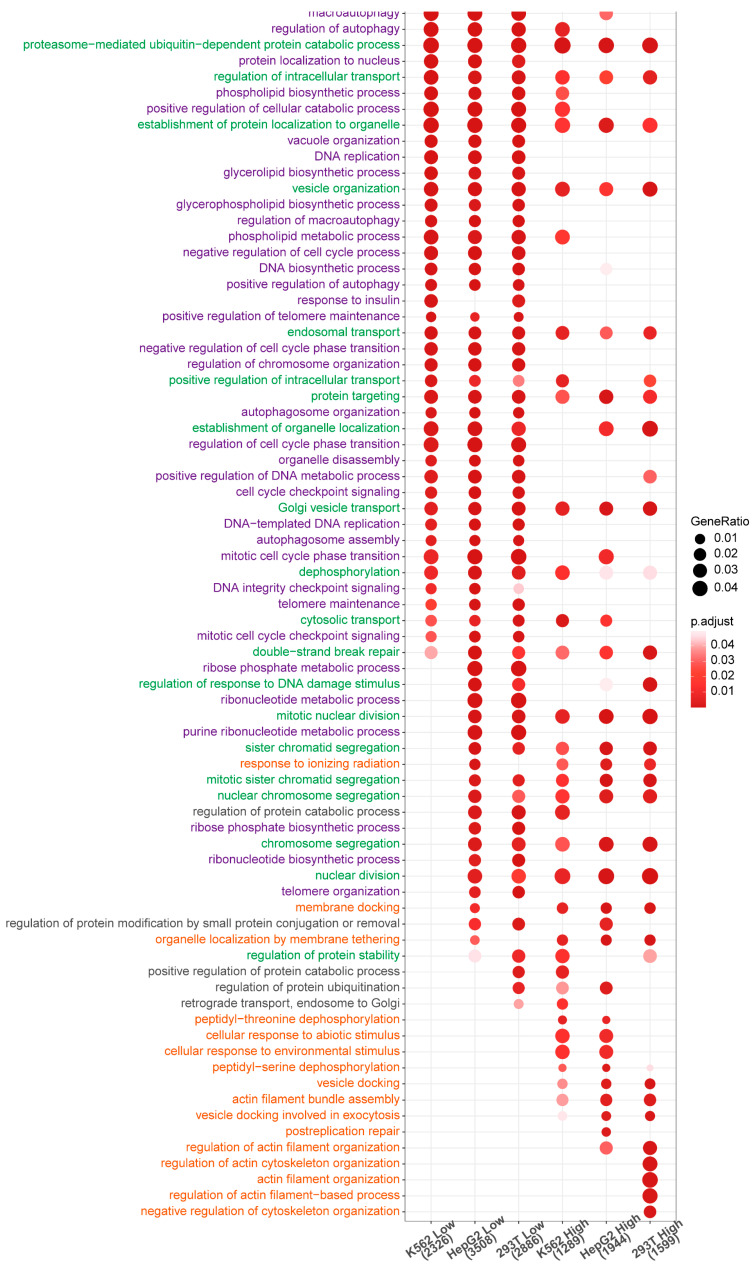
Functional enrichment for genes that are potentially regulated by TSS-proximal eG4s. Genes were grouped by cell line and the stability level of associated eG4s. Biological processes were categorized into three groups based on eG4 stability: those linked to high-stability eG4s (orange), those associated with either high- or low-stability eG4s (green), and those enriched for low-stability eG4s (purple). The genomic annotation as well as the heatmap was generated by clusterProfiler [37].

Gene Ontology enrichment analysis revealed conserved functional clustering across all three cell lines (Figure 6). The associated biological processes could be grouped into three distinct categories, with significant mechanistic implications for cellular states in the cancer (K562 and HepG2) and embryonic kidney cells (HEK293T).

The first category (Figure 6, colored in orange) contained genes potentially regulated by TSS-proximal eG4s with high stability levels. This group suggests a set of genes that mediate the cell’s interaction with its surroundings. Enriched terms related to cytoskeletal dynamics [50] (the terms about “actin cytoskeleton”) and vesicle trafficking [51] (the terms related to “membrane docking,” “vesicle docking,” and “exocytosis”) suggest roles in cell adhesion to the culture substrate (for HepG2 and HEK293T) or cell migration and invasion that are relevant for cancer cells [50,51]. The stress response processes (the terms like “response to ionizing radiation” and “cellular response to abiotic/environmental stimulus”) may reflect adaptation to stresses of the in vitro environment. This group may therefore represent the machinery that allows cells to physically interact with and respond to their local environment.

The second category (Figure 6, colored in green) contained genes associated with either high- or low-stability eG4s. This group represents the core operational machinery of the cell. The functions related to the ubiquitin-proteasome system (the terms like “proteasome-mediated ubiquitin-dependent protein catabolic process” and “regulation of protein stability”) are essential for protein turnover in rapidly dividing cancer cell lines like K562 and transformed lines like HEK293T [52]. The transport of molecules and the organization of organelles (terms such as “Golgi vesicle transport”, “endosomal transport”, and “protein targeting”) are fundamental for the intracellular delivery of molecules and crucial for proper cellular function [53]. The processes associated with cell division (the terms about “mitotic nuclear division”, “chromosome segregation”) and the maintenance of genomic stability (“double-strand break repair”) are critical for cell proliferation [54,55]. Their enrichment across these diverse cell lines suggests that the genes are fundamental “housekeeping” genes supporting basic architecture and division fidelity.

The third category (Figure 6, colored in purple) contained genes associated with low-stability eG4s. This group features stress response, metabolic regulation, and precise cell cycle control. Autophagy-related processes (terms such as “macroautophagy”, “autophagosome organization”) suggest roles in managing cellular resources and clearing damaged components [56]. Anabolic processes (terms such as “phospholipid biosynthesis”, “DNA biosynthetic process”) support rapid proliferation, while “response to insulin” further indicates metabolic signaling involvement. Processes related to cell cycle (“negative regulation of cell cycle process”, “cell cycle checkpoint signaling”, and “telomere maintenance”) suggests a role in the mechanisms enabling immortalization, a hallmark of these cell lines [57,58]. The simultaneous enrichment of anabolic (biosynthesis) and catabolic (autophagy) processes suggests a dynamic regulation of cellular metabolism to support rapid growth under stresses of a cancerous or transformed state.

The functional separation suggests a regulatory division: the first category governs external interface and stress response, while the second and third categories maintain internal homeostasis and proliferation. This implies that TSS-proximal eG4s orchestrate two distinct yet interconnected fundamental capabilities in cancer biology: externally focused stress survival versus internally focused sustained proliferation—through distinct stability-dependent mechanisms.

Notably, stress-response functions preferentially associate with high-stability eG4s (Category 1), suggesting a specialized “inducible switch” mechanism. As rapid adaptation is crucial for survival under acute stress [59], these stable eG4s might serve as quickly formed scaffolds and facilitate prompt chromatin remodeling and coordinated TF recruitment upon stimulation.

Conversely, core machinery irrelative to G4 stability (Category 2) is likely driven by constitutive promoters, where G4s play auxiliary roles, while precise metabolic and checkpoint control (Category 3) might be mediated by low-stability eG4s that possibly act as “rheostats”, allowing for the sensitive, fine-tuned integration of internal signals to optimally manage resources and navigate cell cycle checkpoints.

Together, this dual-mode G4 grammar—an “on/off switch” for external threats and a “dimmer switch with a strong baseline” for internal operations—provides a sophisticated framework for cancer cells to balance environmental adaptation with unrestrained growth.

## 4. Discussion

### 4.1. A Stability-Centric Perspective on G4 Analysis

The central objective of G4 research—mirroring broader biological inquiry—is elucidating cause–effect relationships among variables or events [60]. While traditional approaches mostly rely on controlled experiments, i.e., elaborate molecular experiments about specific G4 loci [8], this study establishes broadly applicable methodological framework for investigating endogenous G4 functionality through the lens of structural stability. Leveraging this framework, we demonstrated the impact of G4 stability on regulation of chromatin and transcription.

Our mapping strategy bridges eG4 regions with quantitative stability metric, as well as other eG4-related signals such as chromatin states, chromatin openness, and TF-binding events, enabling further analysis of the relationships, especially causality between them. Based on the stability-focusing analysis suggested by the framework, we extended the relationships among eG4 stability, eG4 signals, evolutionary conservation, epigenetic marks, chromatin accessibility, and TF binding to causation. The inferred causality—from stability to downstream events—aligns with fundamental biophysical principles, where stability of G4 structures intrinsically impacts molecular interactions and further influences chromatin/transcriptional regulatory processes.

Further functional analysis of TSS-proximal eG4s reveals stability-dependent enrichment patterns: genes downstream of these G4s associate with external interface and stress response (preferentially linked to high-stability eG4s) and internal homeostasis and proliferation (preferentially linked to low-stability eG4s or universally across stability levels). This positions TSS-proximal eG4s as prevailing elements for sustaining the basic processes in survival and growth and suggests a dual-mode G4 grammar. In particular, high-stability eG4s possibly support a genome-wide mechanism, enabling rapid and coordinated regulation of transcriptional responses to external stresses.

While previous genomic studies [11,21,61] have established associations between G-quadruplexes and regulatory regions—including promoters and enhancers—and correlated their presence with various epigenetic marks, our work moves beyond these descriptive observations. The key conceptual advance presented here is that in vitro-derived G4 stability represents an intrinsic structural property that permits causal interpretation of stability-associated genomic patterns. Our study presents the first genome-scale statistical framework for inferring causal relationships between G4 formation and cellular processes. By mapping the genome-wide G4 stability landscape, we provide a missing explanatory dimension for why certain G4 loci coincide with downstream regulatory events, such as strong epigenetic modifications and transcription factor occupancy, while others do not. This stability-centric principle not only provides a unifying mechanistic hypothesis for previously fragmented observations but also shifts the paradigm from cataloging correlations to building predictive models of G4 function.

### 4.2. Limitations and Future Directions

While our study establishes a robust statistical framework linking G4 thermostability to functional genomic outcomes, we acknowledge the limitations of our computational approach, which also illuminate productive avenues for future research.

First, while the acyclic structure of Bayesian networks is necessary for causal discovery, it may not fully capture the feedback mechanisms often present in biological pathways. For instance, although our model strongly supports the causal path from chromatin accessibility to histone modification states, we acknowledge that bidirectional regulatory relationships likely exist, where histone modifications may also influence chromatin openness. Such reciprocal effects remain challenging to resolve within our current computational framework.

Second, the resolution of our multi-omics integration is naturally constrained by the characteristics of bulk sequencing technologies. The signals we analyze represent population-level averages, which may mask the single-cell heterogeneity in G4 formation and function. Additionally, our mapping strategy assigns a single stability value to each eG4 region, which might not fully capture the dynamic nature and structural diversity of G4 conformations at individual loci across different cells.

Third, like most current high-throughput G4 mapping approaches, our method cannot systematically distinguish between intramolecular and intermolecular G4 topologies. While existing evidence suggests that intramolecular structures predominate in chromatin environments, future methodological advances in structural sequencing will be crucial for elucidating how specific topological states contribute to the stability–function relationships identified here.

Furthermore, important challenges remain in elucidating G4-mediated regulatory mechanisms. Future research would benefit from the incorporation of quantitative causal modeling with expanded datasets encompassing additional epigenetic marks, while accounting for technical covariates such as unobserved factors influencing chromatin states and potential artifacts from sample processing and sequencing. Investigating G4 dynamics at individual loci would provide crucial insights into their conformational transitions during cellular processes, particularly when integrated with structural databases such as G4Atlas [62] and ONQUADRO [63], which document experimentally determined G4 conformations and their sequence–structure relationships. The adaptation of single-cell multi-omics approaches, such as G4-miner [10] for single-cell genomics, could effectively resolve cell-to-cell heterogeneity in G4 formation and function. Additionally, well-designed molecular experiments remain essential for validating the specific mechanisms through which G4 structures participate in stress adaptation processes. Together, these complementary approaches would help bridge the gap between computational predictions and mechanistic understanding of G4 biology.

## Figures and Tables

**Table 1:** Datasets used in the study.

Cell Line	Data	Source	Comment
K562	G4 ChIP-Seq, with BG4, Single-end	GEO: GSE145090	G4 peak regions
	ATAC-Seq	ENCODE: ENCSR483RKN	For analysis of chromatin openness signal
	chromeState	The NIH Roadmap Epigenomics, Expanded 18-state model: https://egg2.wustl.edu/roadmap/data/byFileType/chromhmmSegmentations/ChmmModels/core_K27ac/jointModel/final/ (accessed on 1 January 2025)	For analysis of chromatin state
	TF binding	The ReMap2022 datasets: https://remap.univ-amu.fr/storage/remap2022/hg19/MACS2/remap2022_all_macs2_hg19_v1_0.bed.gz (accessed on 1 January 2025)	For analysis of TF- binding events
HepG2	G4 ChIP-Seq, with BG4, Single-end	GEO: GSE145090	G4 peak regions
	ATAC-Seq	GEO: GSE170251 ENCLB750JRI	For analysis of chromatin openness signal
	chromeState	The NIH Roadmap Epigenomics, Expanded 18-state model: https://egg2.wustl.edu/roadmap/data/byFileType/chromhmmSegmentations/ChmmModels/core_K27ac/jointModel/final/ (accessed on 1 January 2025)	For analysis of chromatin state
	TF binding	The ReMap2022 datasets: https://remap.univ-amu.fr/storage/remap2022/hg19/MACS2/remap2022_all_macs2_hg19_v1_0.bed.gz (accessed on 1 January 2025)	For analysis of TF- binding events
HEK293T	G4 ChIP-Seq, with BG4, Paired-end	GEO: GSE178668	G4 peak regions
	ATAC-Seq	GEO: GSE235014	For analysis of chromatin openness signal
	H3K4me1	GEO: GSE178668	For prediction and analysis of the cell-specific chromatin state
	H3K4me3	GEO: GSE178668	
	H3K9me3	GEO: GSE208200	
	H3K27ac	GEO: GSE178668	
	H3K27me3	GEO: GSE235014	
	H3K36me3	GEO: GSE147752	
	chromeState	The NIH Roadmap Epigenomics, Expanded 18-state model: https://egg2.wustl.edu/roadmap/data/byFileType/chromhmmSegmentations/ChmmModels/core_K27ac/jointModel/final/ (accessed on 1 January 2025)	For analysis of chromatin state
	TF binding	The ReMap2022 datasets: https://remap.univ-amu.fr/storage/remap2022/hg19/MACS2/remap2022_all_macs2_hg19_v1_0.bed.gz (accessed on 1 January 2025)	For analysis of TF- binding events
Non-cell-specific data	pG4 motifs	pqsfinder: https://pqsfinder.fi.muni.cz/hub/hg19/pqsfinder_hg19_gff.tar.gz (accessed on 1 January 2025)	Putative G4 loci
	G4-Seq	GEO: GSE63874	G4 stability data (Mismatch Percentage)
	phyloP score	Zoonomia Project: https://cgl.gi.ucsc.edu/data/cactus/241-mammalian-2020v2-hub/Homo_sapiens/241-mammalian-2020v2.bigWig (accessed on 1 January 2025)	Evolutionary conservation data

## Data Availability

The prepared datasets for the three cell lines and the codes that support the findings of this study are openly available in GitHub at https://github.com/kexiao-nj/G4StabilityAnalysis (accessed on 9 September 2025).

## Supplementary Materials

The following supporting information can be downloaded at: https://www.mdpi.com/article/10.3390/genes16101231/s1, Figure S1: The enrichment of each state for a set of external genomic annotation, and at fixed positions relative to TSS and TES; Figure S2: Distribution of average phyloP score at the pG4 sites; Figure S3: The coverage and accuracy of models derived from different strategies and sample sizes; Figure S4: The common Bayesian networks derived from different strategies and sample sizes; Table S1: Dataset prepared for K562; Table S2: Dataset prepared for HepG2; Table S3: Dataset prepared for HEK293T; Table S4: The adjacency matrix of the Bayesian network about eG4Sig, Stability, phyloP, ATACSig, ChromState, and #TFs.
