# Supplementary material for: Stability Matters: Revealing Causal Roles of G-Quadruplexes (G4s) in Regulation of Chromatin and Transcription
Source: Genes (Basel). 2025 Oct 17;16(10):1231. doi: 10.3390/genes16101231 (PMC12564469; doi:10.3390/genes16101231)
Supplement: Supplementary file 1 [file genes-16-01231-s001.zip › Supplementary Figures.pdf]

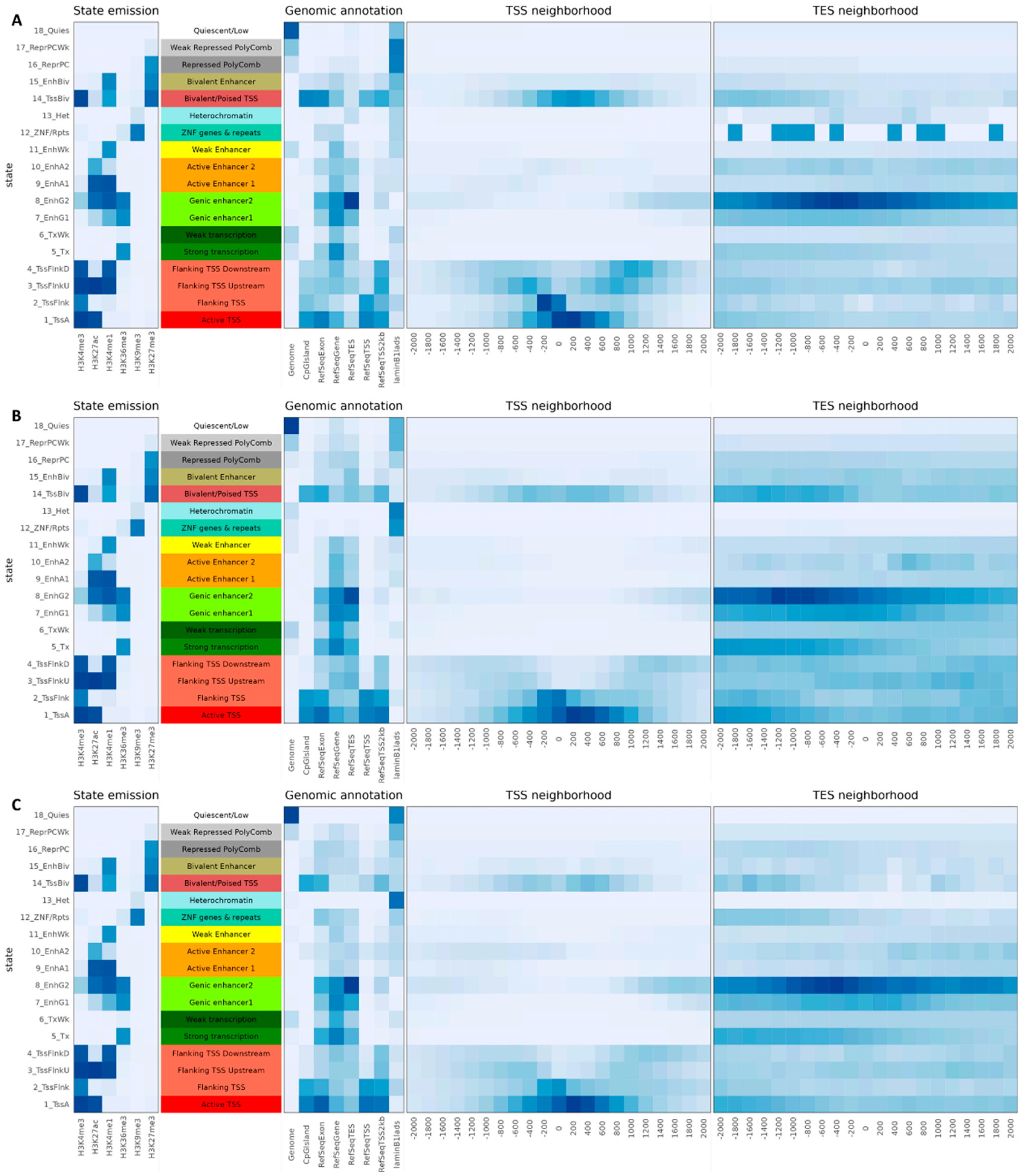

Figure S1. The enrichment of each state for a set of external genomic anntation, and at fixed positions relative to TSS and TES. (A) HEK293T. (B) HepG2. (C) K562.

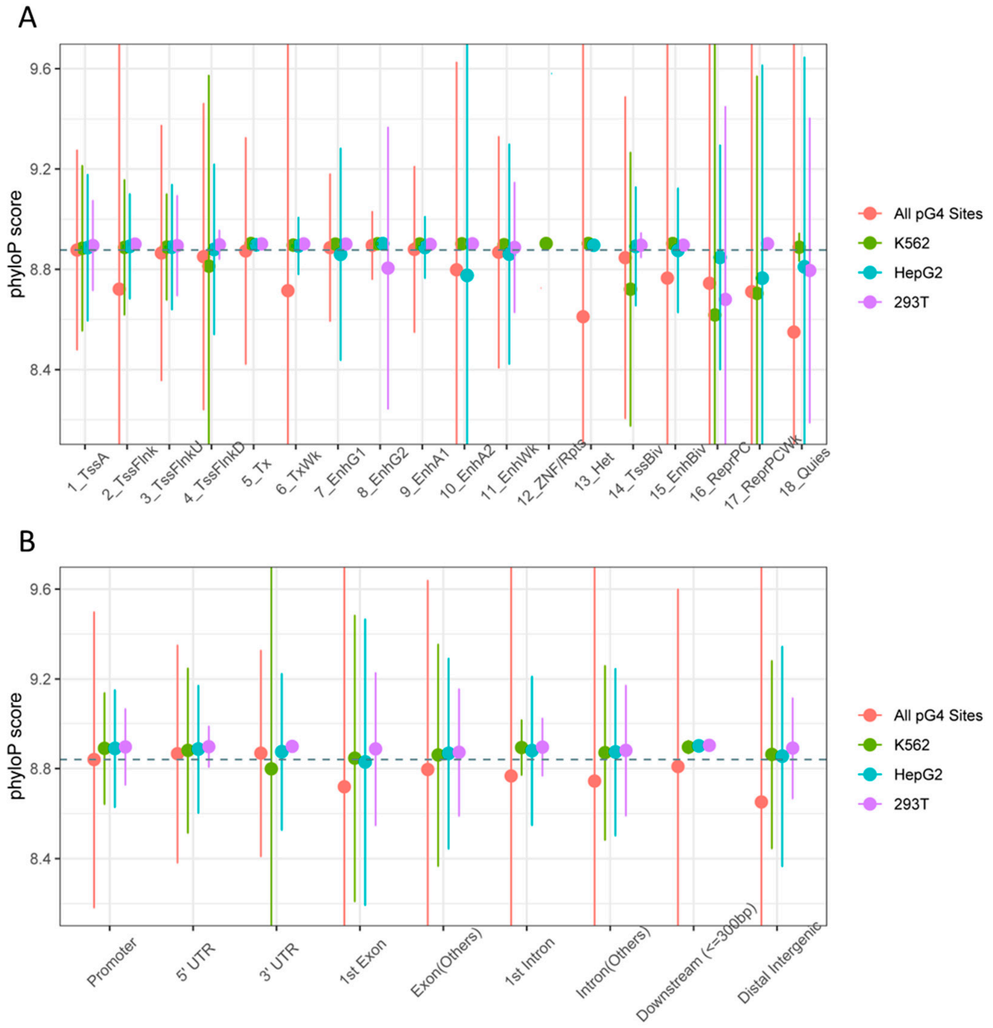

Figure S2. Distribution of average phyloP score at the pG4 sites. The dots inicate the mean values, and the bars indicate the standard errors. (A) Distribution of phyloP score across different chromatin states. The states were defiend as in Figure 3 and 1S. (B) Distribution of phyloP score across different genomic regions.

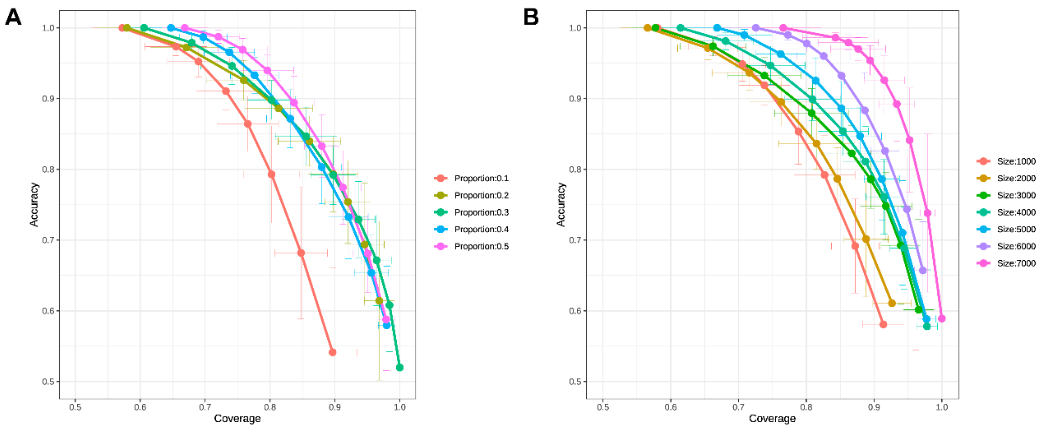

Figure S3. The coverage and accuracy of models derived from different strategies and sample sizes. (A) Models derived from proportional-allocation strategies (extracting 10%, 20%, 30%, 40%, and 50% samples of each stratum respectively). (B) Models derived from equal-allocation strategies (extracting 1000, 2000, 3000, 4000, 5000, 6000, and 7000 samples from each stratum respectively). For each N (an integer from 1 to 10), model accuracy and coverage were calculated to generate the performance curve for each sampling strategy. As the common-network-generating process were repeated 50 times, the coverage and accuracy at each point is the average of 50 trials. The vertical and horizontal bars on the curves indicate the standard deviations of accuracy and coverage.

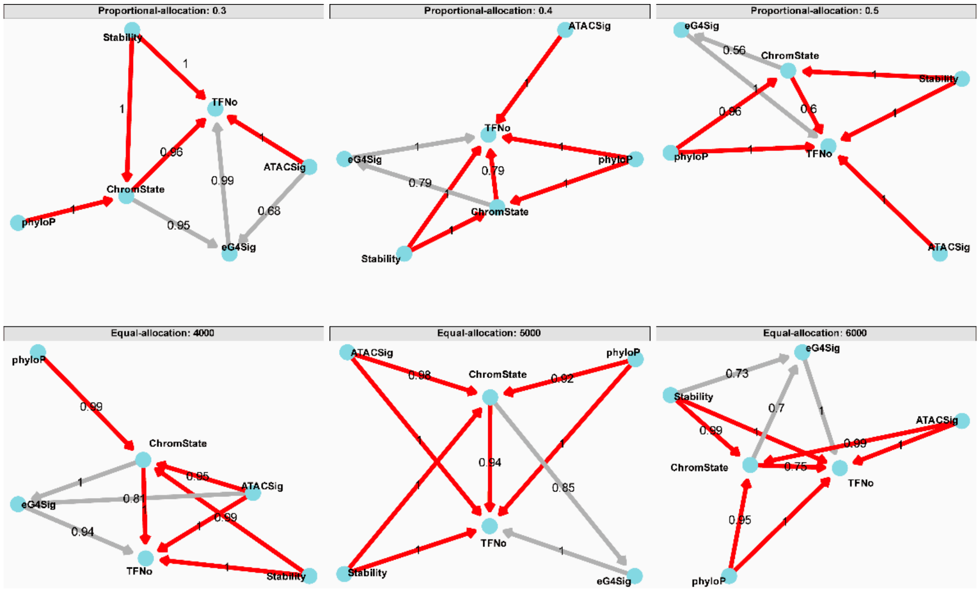

Figure S4. The common Bayesian networks derived from different strategies and sample sizes. The networks were generated by the proportional-allocation and equal-allocation strategies, respectively extracting 40%, 50%, 60%, 4000, 5000 and 6000 samples from each stratum. The numbers indicate the direction probability of each edge. The proposed causality Stability/phyloP/ATACSig→{ChromState, #TFs} as well as ChromState→#TFs were indicated by red edges.
